# Dental anxiety and oral health following stroke: a pilot study

**DOI:** 10.1186/s12903-022-02618-z

**Published:** 2022-12-03

**Authors:** Matthew R. Nangle, Alexandra G. Adams, Julie D. Henry

**Affiliations:** 1 School of Dentistry, The University of Queensland, Brisbane, QLD Australia; 2 School of Psychological Sciences, The University of Newcastle, Newcastle, NSW Australia; 3 School of Psychology, The University of Queensland, Brisbane, QLD Australia

**Keywords:** Older adults, Oral health, Dental anxiety, Stroke, Burden of disease

## Abstract

**Background:**

Oral health is often poorer in people living with acquired brain injury relative to non-clinical controls. However, although anxiety disorders become more common following stroke, no study to date has tested whether dental anxiety might contribute to stroke survivors’ increased vulnerability to poorer oral health. This pilot study reports the first test of whether the anxiety disturbances that commonly present following stroke extend to dental anxiety, and if dental anxiety in this group is linked to poorer oral health.

**Materials and methods:**

First-time stroke survivors (N = 35) and demographically matched controls (N = 35) completed validated measures of dental anxiety, oral health, negative affect, and life satisfaction.

**Results:**

Stroke survivors did not differ from controls in their overall levels of dental anxiety or oral health, but uniquely for the stroke group, dental anxiety was strongly associated with poorer oral health, and this effect remained significant even after controlling for negative affect and life satisfaction.

**Conclusion:**

Stroke survivors who have higher levels of dental-related anxiety may be at increased risk of poorer oral health.

## Background

Stroke is a serious cerebrovascular condition caused by an interruption of blood supply to the brain. This interruption can happen in two main ways: via a blockage (ischaemic stroke) or a bleed (haemorrhagic stroke). Both are potentially devastating, with stroke a major cause of mortality and morbidity globally. Although stroke can occur at any age, aging is the most robust non-modifiable risk factor, with risk approximately doubling for each subsequent decade of life after the age of 55 [1]. With the world’s population aging at an unprecedented rate, the number of people surviving with stroke-related disability has been projected to continue increasing. For instance, in Europe there were 9.53 million stroke survivors in 2017, and modelling indicates that this number will increase a further 27% by 2047 [2].

Given such statistics, it is unsurprising that considerable focus is now being placed on how to improve quality of life following stroke. Many of the risk factors for stroke (such as being overweight, heavy alcohol consumption, oral diseases, and diabetes) can impact wellbeing. Yet often underappreciated in this literature is the fundamental role of oral health for broader wellbeing. Poor oral health can cause pain and infection, with major consequences to quality of life as well as broader physical and mental health [3]. People whose facial appearance or speech are impaired by oral diseases are also more likely to avoid social interactions owing to concerns over how they look and their ability to communicate. These isolating effects have negative consequences for broader mental and physical health, with loneliness and social isolation now recognised as a priority public health problem and policy issue for older people [4].

Importantly, both the cognitive and physical losses associated with stroke have the potential to negatively impact oral health via behavioral pathways, such as through reducing the ability to effectively engage in oral hygiene or to access dental health services. Consistent with this possibility, a systematic review recently revealed that oral health is poorer in patients with acquired brain injury (ABI) relative to non-clinical controls, with most of the studies contributing to this review focused on stroke survivors specifically [5]. Stroke survivors therefore appear to be at heightened risk of poor oral health.

The aim of this pilot study was to provide the first test of whether dental anxiety in this clinical group might be related to their increased vulnerability to poorer oral health. This question is of interest because, not only do anxiety disorders become more common following stroke, estimated to affect 29.3% of stroke survivors in the first-year post-stroke [6], but a prospective cohort study revealed that the nature of this disturbance is predominantly phobic in presentation [7]. Phobic anxiety is characterized by an excessive and irrational fear of a particular object, activity, or situation, accompanied by marked avoidant behaviour of specific anxiety-provoking situations. One of the ways in which phobic anxiety can present is as dentophobia (dental fear and anxiety), and a recent review concluded that approximately 20% of adults in the general population experience moderate dentophobia [8]. Because people with dental fear and anxiety are more likely to avoid the dentist and neglect oral hygiene, people with high levels of dentophobia are also more vulnerable to poor oral health.

This pilot study will integrate these previously disparate literatures by providing the first test of the predictions, that (1) anxiety disturbances during the first year post-stroke also extend to dental anxiety, and (2) greater dental anxiety in stroke survivors will be associated with poorer oral health. The study will also test whether the predicted relationship between oral health and anxiety remains significant after adjusting for negative affect and wellbeing, to establish specificity.

## Methods

The primary dependent measure of interest in this study was the measure of dental anxiety, the Index of Dental Anxiety and Fear [IDAF-4 C; 10]. Power calculations were based on sensitivity to detect between-group effects and within-group correlations on the IDAF-4 C that were large in magnitude. A formal power analysis using G*Power 3.1 software revealed that, for the between-group comparisons, the minimum number of participants required to detect group differences that were large in magnitude (as indexed by the standardized mean difference Cohen’s *d* = 0.80, one of the most common ways to measure effect size) at conventionally accepted power levels (80%) was 26 participants per group. For the correlational analyses, to have this same level of power to detect large-sized correlations (*r* = 0.50), G*Power also showed a total of 26 participants were required. In the current study we therefore sought to recruit enough participants in each group to be well above this cut-off.

After providing written, informed consent, a total of 70 participants took part: 35 who had suffered a first-time stroke and 35 demographically matched controls. Stroke participants were deemed eligible if they had suffered a first-time stroke, as confirmed via MRI or CT, between 9 and 18 months prior to testing. All stroke participants were recruited following admission to either the Princess Alexandra Hospital or the Royal Brisbane and Women’s Hospital in Brisbane, or via a retrospective clinical file audit at The Mater Hospital. All control participants were recruited via community-based advertising. Inclusion criteria for both groups also included no current diagnosis or history of any neurological disorder or serious psychiatric illness (other than stroke for those in the stroke group). All control participants aged over 65 and over also completed a cognitive screening tool for dementia. No participant who presented for testing from either group had to be excluded based on any of the above criteria. To be eligible, all participants had to be a native English speaker, or have a high level of English proficiency.

## Measures

Participants in this study also completed measures for an unrelated published study [9]. All participants completed the assessments in individualised face-to-face testing sessions. To minimise potential order effects, all assessments in the complete testing protocol were divided into two blocks with order of presentation counterbalanced across participants (and individual measures also counterbalanced within each of these blocks). Only measures relevant to the current study are reported here. All measures were selected because they have been extensively validated as indicators of the constructs they were designed to assess.

The 8-item base module of the Index of Dental Anxiety and Fear [IDAF-4 C; 10] was used to assess dental anxiety and fear. The eight questions index emotional, behavioural, physiological, and cognitive components of dental fear. This measure has excellent psychometric properties and is predictive of future dental service use and avoidance due to fear. Each item is scored between 1 (disagree) and 5 (strongly agree), with higher scores indicative of greater dental anxiety.

Oral health was assessed using the Oral Health Questionnaire [OHQ; 11]. The OHQ includes ten items that were identified as being most predictive of the World Health Organisation (WHO) diagnostic criteria for decayed, missing and filled teeth [11]. The items provide data relating to oral hygiene habits, use of oral health services, smoking status, and disease presence (bleeding gums, mobility, decay, orofacial pain, and halitosis). The total score ranges from 0 to 20, with higher OHQ scores indicative of poorer oral health.

Negative affect was indexed using The Hospital Anxiety and Depression Scale [HADS; 12]. The HADS is an extensively used self-report scale with sound psychometric properties. It is particularly suitable for use in clinical populations as it omits somatic items that might be attributable to physical illness. The HADS consists of 14 items, seven of which measure anxiety, the other seven depression. Scoring for each item ranged from zero to three, with a sum score range of 0–42. Higher scores are indicative of greater negative affect.

Quality of life was assessed using the World Health Organisation Quality of Life Scale [WHO QOL-BREF; 13], an extensively validated 26-item self-report measure, that indexes four domains: physical health (seven items), psychological health (six items), social relations (three items), and environment (eight items). The remaining two items provide an overall rating of subjective satisfaction with health, and quality of life. Scores for all four domains are summed, with a higher total score indicative of higher quality of life.

## Analysis

The clinical and control groups were compared on the measures of dental anxiety, oral health, negative affect, and wellbeing using a series of independent samples t-tests. In instances where violations of the sphericity assumption occurred, degrees of freedom and p-values were adjusted using Greenhouse–Geisser corrections. Correlational and partial correlations were computed to test correlates of dental anxiety in the stroke and control groups separately. SPSS (version 27.0) was used for all analyses.

## Results

The stroke and control groups were matched in terms of gender composition (both 56.7% male), and were closely matched in terms of age (M = 64.69, SD = 12.92 and M = 63.23, SD = 9.75, respectively, t(68) = 0.53, *p* = 0.596) as well as years of education, M = 14.09, SD = 3.72 and M = 14.64, SD = 2.69, respectively, t(68) = 0.72, *p* = 0.476).

Descriptive statistics for all measures are reported in Table 1. Formal analysis of the four key dependent measures of interest were conducted (for which Bonferroni corrections yielded an adjusted p-value of 0.0125) and revealed that the two groups did not differ with respect to dental anxiety, oral health, negative affect, or quality of life.

**Table 1:** Scores on the measures of dental anxiety, oral health, negative affect and quality of life

Measure	Control (N = 35)			Stroke (N = 35)			t-tests		
	M	SD	Range	M	SD	Range	t	df	*p*
IDAF	1.81	1.04	1–5	1.78	1.08	1–5	0.14	68	0.891
OHQ	5.20	3.29	0–16	4.30	2.92	0–13	1.19	68	0.240
HADS	9.17	4.50	2–18	11.38	5.89	3–23	1.75	68	0.084
WHO QOL-BREF	62.65	8.04	48–78	63.65	6.67	44–79	0.53	68	0.599

*IDAF* Index of Dental Anxiety and Fear, *OHQ* Oral Health Questionnaire, *HADS* Hospital Anxiety and Depression Scale, *WHO QOL-BREF* World Health Organization Quality of Life Scale-Brief

Pearson product-moment correlations were then conducted to test the relationships between dental anxiety with oral health, negative affect, and quality of life (see Table 2). After applying Bonferroni corrections (which yielded an adjusted p-value of 0.008), these analyses revealed that in the control group dental anxiety was not related to any of the measures. However, in the stroke group, higher levels of dental anxiety were strongly and significantly associated with poorer oral health (r = 0.54, *p*  < 0.001). Significant and comparable sized associations also emerged between dental anxiety with both quality of life and negative affect (rs = − 0.47 and 0.51, ps = 0.006 and 0.002, respectively). However, a partial correlation that controlled for quality of life and negative affect to establish the specificity of the association between dental anxiety and oral health revealed an effect that remained significant, and which was almost identical in size to the raw correlation (r_p_ = 0.52, *p* = 0.002).

**Table 2:** Correlations between dental anxiety with oral health, negative affect, and quality of life, calculated separately for the two groups

	OHQ	HADS	WHO-QOL BREF
*Control group*			
IDAF	0.16	0.04	− 0.24
*Stroke group*			
IDAF	0.54*	0.51*	-0.47*

*IDAF* Index of Dental Anxiety and Fear, *OHQ* Oral Health Questionnaire, *HADS* Hospital Anxiety and Depression Scale, *WHO QOL-BREF* World Health Organization Quality of Life Scale-Brief

*p < Bonferroni adjusted value of 0.008

## Discussion

These data provide novel evidence about dental anxiety and oral health in first-time stroke survivors. The key finding to emerge was that, although there was no evidence of poorer oral health or increased dental anxiety up to 18 months following stroke, a higher level of dental anxiety was significantly and substantially correlated with poorer oral health in this group.

As noted previously, it had been anticipated that stroke survivors would have poorer oral health given that a recent systematic review concluded that oral health problems are more common following acquired brain damage [5]. However, in many of the studies contributing to this review, microbiological or biochemical aspects of oral health were assessed, and these are more sensitive to early deterioration than the self-report measure used here. Additionally, many of the contributing studies were completed in clinical care environments and included stroke survivors who had a longer chronicity and/or who had suffered severe, or recurrent strokes. A key conclusion of Kothari et al.’s [5] review was that hospitalisation itself was linked to more deteriorated oral health. Perhaps most importantly, many of the oral health problems indexed by the measure used here (such as tooth mobility and tooth cavities) only occur after a lengthy period of deterioration. Not only were all the stroke participants that contributed to the present study first-time stroke survivors who had returned to their own homes, but all were also tested only approximately one year following this initial stroke (average chronicity was 13.64 months, SD = 2.29).

Most importantly here, although overall stroke survivors did not differ from controls in their degree of dental anxiety, the stroke (but not the control group’s) level of dental anxiety was significantly correlated with their oral health. Indeed, the magnitude of this effect qualified as a large effect even after covarying for broader negative affect and satisfaction with life. This indicates that it is dental anxiety specifically and not broader psychopathology that is related to oral health following stroke. It also provides preliminary support for the possibility that, even when dental anxiety is no more frequent in this clinical cohort than in non-clinical controls, when it does present it may have more serious consequences.

Broader literature shows how dental anxiety can, over time, establish a vicious cycle, whereby the initial avoidance of dental treatment may lead to treatment being delayed until the point that dental pain can no longer be endured - at which point the oral health deterioration may have become so severe that more invasive, painful treatment is required. This then leads to a reinforcement of dental fear, and future avoidance of treatment-seeking. [8, 10]. Although dental anxiety would therefore be of concern in any group, the present study suggests that it may be particularly problematic following stroke. It is cautiously suggested that this may be because broader losses in both cognitive and physical function that are caused by stroke make it more challenging to engage in good oral hygiene and to access external oral health care. Dental anxiety may therefore be especially problematic since it will presumably reduce motivation to try and overcome these other challenges. Future work is now needed to directly test this possibility using longitudinal research methods, and to also see if the strong relationship between dental anxiety and oral health identified following stroke is also evident in other clinical groups that present with chronic cognitive and/or physical limitations.

Finally, some limitations need to be acknowledged. First, although the present study was adequately powered to detect largesized effects, it was underpowered to detect weaker ones, and moving forward larger sized samples are therefore important. Related to this point, because of the sample size, we treated stroke here as a single homogeneous group, yet there are many differences between stroke survivors that might potentially influence their vulnerability to dental anxiety as well as oral health. This includes not only clinical characteristics of the stroke (type, location, and size), but also factors that influence recovery (such as support networks, and financial resources). Future research should also seek to consider this broader heterogeneity in trying to understand the relationship between dental anxiety and oral health following stroke.

## Conclusion

Dental anxiety has important individual, clinical, and public health consequences. Although oral health is often assigned a relatively low priority in clinical care, these pilot data provide initial evidence that routine screening for dental anxiety post-stroke might potentially prevent future deterioration of oral health, and therefore contribute to improved overall wellbeing.

## Data Availability

The de-identified data that support the findings of this study are available from the corresponding author upon reasonable request.
